# Antitumor Activity and Physicochemical Properties of New Thiosemicarbazide Derivative and Its Co(II), Ni(II), Cu(II), Zn(II) and Cd(II) Complexes

**DOI:** 10.3390/molecules27092703

**Published:** 2022-04-22

**Authors:** Bartłomiej Rogalewicz, Alina Climova, Ekaterina Pivovarova, Jarosław Sukiennik, Kamila Czarnecka, Paweł Szymański, Małgorzata Szczesio, Katarzyna Gas, Maciej Sawicki, Monika Pitucha, Agnieszka Czylkowska

**Affiliations:** 1 Institute of General and Ecological Chemistry, Faculty of Chemistry, Lodz University of Technology, Zeromskiego 116, 90-924 Lodz, Poland; alina.climova@dokt.p.lodz.pl (A.C.); ekaterina.pivovarova@dokt.p.lodz.pl (E.P.); jaroslaw.sukiennik@dokt.p.lodz.pl (J.S.); malgorzata.szczesio@p.lodz.pl (M.S.); 2 Department of Pharmaceutical Chemistry, Drug Analyses and Radiopharmacy, Faculty of Pharmacy, Medical University of Lodz, Muszyńskiego 1, 90-151 Lodz, Poland; kamila.czarnecka@umed.lodz.pl (K.C.); pawel.szymanski@umed.lodz.pl (P.S.); 3 Department of Radiobiology and Radiation Protection, Military Institute of Hygiene and Epidemiology, 4 Kozielska St., 01-163 Warsaw, Poland; 4 Institute of Physics, Polish Academy of Sciences, Aleja Lotnikow 32/46, PL-02668 Warsaw, Poland; kgas@ifpan.edu.pl (K.G.); mikes@ifpan.edu.pl (M.S.); 5 Independent Radiopharmacy Unit, Faculty of Pharmacy, Medical University of Lublin, PL-20093 Lublin, Poland; monika.pitucha@umlub.pl

**Keywords:** thiosemicarbazides, metal complexes, A549 cancer cells, antitumor activity, biological activity, thermogravimetric analysis, spectroscopic properties, magnetic properties

## Abstract

A novel biologically active thiosemicarbazide derivative ligand *L* (*N-[(phenylcarbamothioyl)amino]pyridine-3-carboxamide*) and a series of its five metal(II) complexes, namely: [Co(L)Cl_2_], [Ni(L)Cl_2_(H_2_O)], [Cu(L)Cl_2_(H_2_O)], [Zn(L)Cl_2_] and [Cd(L)Cl_2_(H_2_O)] have been synthesized and thoroughly investigated. The physicochemical characterization of the newly obtained compounds has been performed using appropriate analytical techniques, such as ^1^H and ^l3^C nuclear magnetic resonance (NMR), inductively coupled plasma (ICP), thermogravimetric analysis (TGA), Fourier-transform infrared spectroscopy (FTIR) and magnetic measurements. In order to study the pharmacokinetic profile of the compounds, ADMET analysis was performed. The in vitro studies revealed that the synthesized compounds exhibit potent biological activity against A549 human cancer cell line.

## 1. Introduction

**Figure 1:**
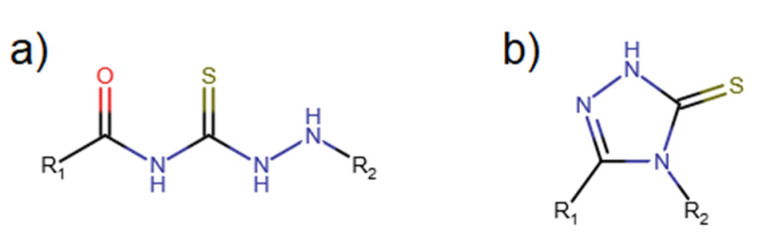
Thiosemicarbazides (**a**) and 1,2,4-triazol-3-thiones (**b**).

The process of the search of new drugs is a challenge which involves the participation of different research laboratories and medical groups. Well-known and described diseases change their form and improve their endurance against medicines, which become less and less effective. In that continuous race for the new active compounds, or the increase in the activity of present structures, our research team contributes to the development of new effective drugs. Thiosemicarbazides (Figure 1a) are known to exhibit antitubercular [1,2] antibacterial [3,4] and herbicidal properties [5]. There are reported derivatives with tuberculostatic activity [6] and against large spectra DNA and RNA viruses [7,8,9]. They have promising anticancer properties [10]. Additionally, complexes of thiosemicarbazides with metals ions can increase their activity [11,12].

**Figure 2:**
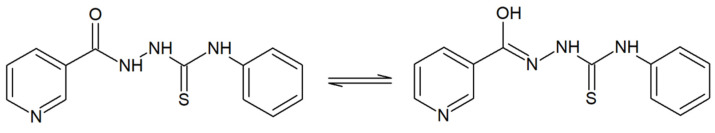
Tautomers of the obtained ligand, *N-[(phenylcarbamothioyl)amino]pyridine-3-carboxamide*.

Based on the thiosemicarbazides, 1,2,4-triazol-3-thiones (Figure 1b) can be synthesized [12]. Their derivatives are promising compound agents against viruses and a few cancers [13,14]. The formation of complexes of active compounds using metal ions as coordinate centers is one of the methods which can increase their properties as active molecules. It is possible to manipulate the compound’s activity by changing the metal ion in the complex [15]. One of the most popular metal ion used in these kind of research is copper (II), which itself has biological activity [16] such as blood–brain barrier penetration. Excess of copper in the human organism can cause Alzheimer’s Disease [17]. **C**opper complexes have numerous properties including proteasome activity inhibitors [18], DNA intercalation [19] and anticancer chelators. They can also increase the activity of the ligand itself. For example, *4-Cyclohexyl-3-(4-nitrophenyl)methyl-1,2,4-triazolin-5-thione* has no activity against selected bacteria, but after obtaining the metal(II) complex, the activity increases to a mild score [20]. A few of the subjects of our studies are the thiosemicarbazide derivatives, such as the one presented here, *N-[(phenylcarbamothioyl)amino]pyridine-3-carboxamide* (Figure 2). These kinds of organic compounds are known in the literature as substrates for the synthesis of heterocyclic compounds [21,22].

In recent years, scientists have become more interested in the biological activity of thiosemicarbazide derivatives. Some of them have an antiproliferative effect on the human breast adenocarcinoma and human hepatocellular carcinoma cell lines [23]. Compounds are known to significantly inhibit A549, HepG2 and MCF-7 cell division [24]. *1-(Pyridin-3-yl)-4-(phenyl)thiosemicarbazide* was screened for urease inhibitory [25]. In our work we used the obtained compound for a series of the complexation reactions using Co(II), Ni(II), Cu(II), Zn(II) and Cd(II) chloride salts. We present our study of the synthesis procedure and physicochemical properties, as well as biological activity investigations of the synthesized compounds. They were assessed as cytotoxic candidates against human lung carcinoma cells (A549).

## 2. Results and Discussion

### 2.1. Thermogravimetric Analysis (TGA) Analysis

**Figure 3:**
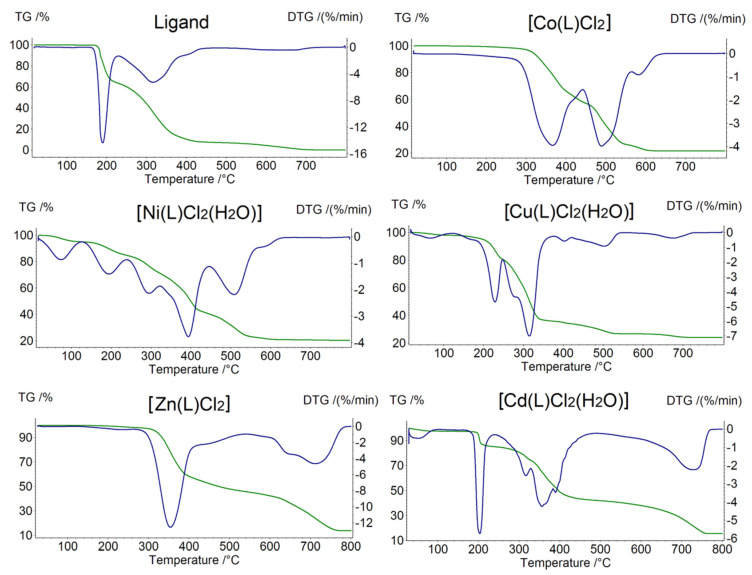
TG (green)-DTG (blue) curves of the free ligand and five complexes.

Thermogravimetric analysis (TGA) is an alternative, fast and useful technique for determining composition of coordination compounds. Therefore, thermograms of the ligand and five complexes: [Co(L)Cl_2_], [Ni(L)Cl_2_(H_2_O)], [Cu(L)Cl_2_(H_2_O)], [Zn(L)Cl_2_] and [Cd(L)Cl_2_(H_2_O)] were prepared and analyzed (Figure 3). Table 1 demonstrates the decomposition stages of the complexes with the identified temperature ranges. All compounds decompose progressively. Hydrous complexes of Ni(II), Cu(II) and Zn(II) are stable up to 40 °C, while anhydrous Co(II) and Zn(II) is up to 280 °C. Thermolysis of free ligand starts at 180 °C. Based on the obtained results, we are able to distinguish general decomposition stages: two in Zn(II) complex; three in free ligand, Co(II), Cd(II) compounds; and four in the case of Ni(II) and Cu(II). The first stage of decomposition of the Cd(II), Cu(II) and Ni(II) complexes clearly proves the presence of H_2_O molecule in these compounds. The next stages are connected with partial and total decomposition of the organic ligand. As the temperature increases, the anhydrous Co(II) and Zn(II) complexes undergo gradual thermal decomposition combined with the destruction of the organic ligand. The last stage for all coordination compounds is the decomposition of the residual parts of the organic ligand, loss of chloride anions and the formation of pure metal oxides. Pyrolysis of free ligand stops around 700 °C with total decomposition.

### 2.2. Fourier-Transform Infrared Spectroscopy (FTIR) Analysis

**Figure 4:**
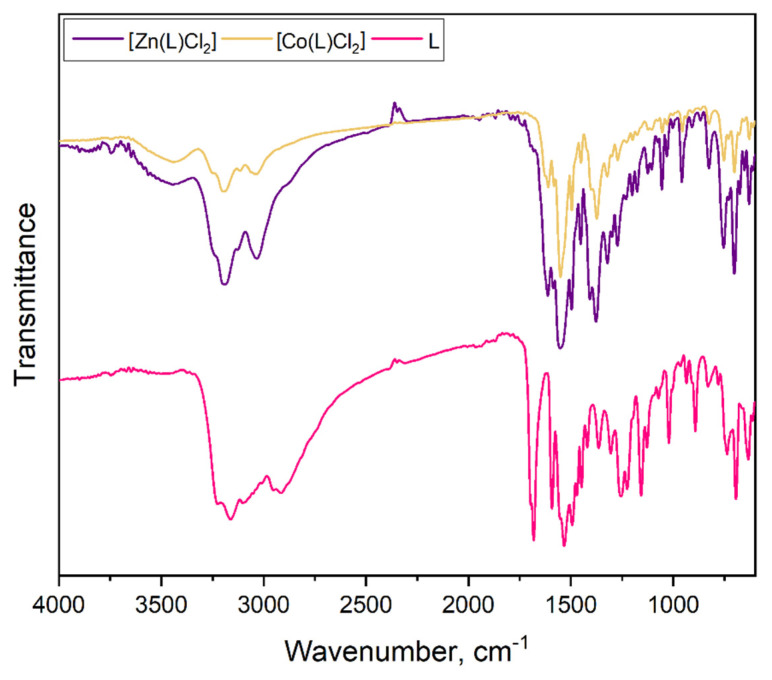
FTIR spectra of pure ligand and obtained complexes of [Co(L)Cl_2_] and [Zn(L)Cl_2_].

**Figure 5:**
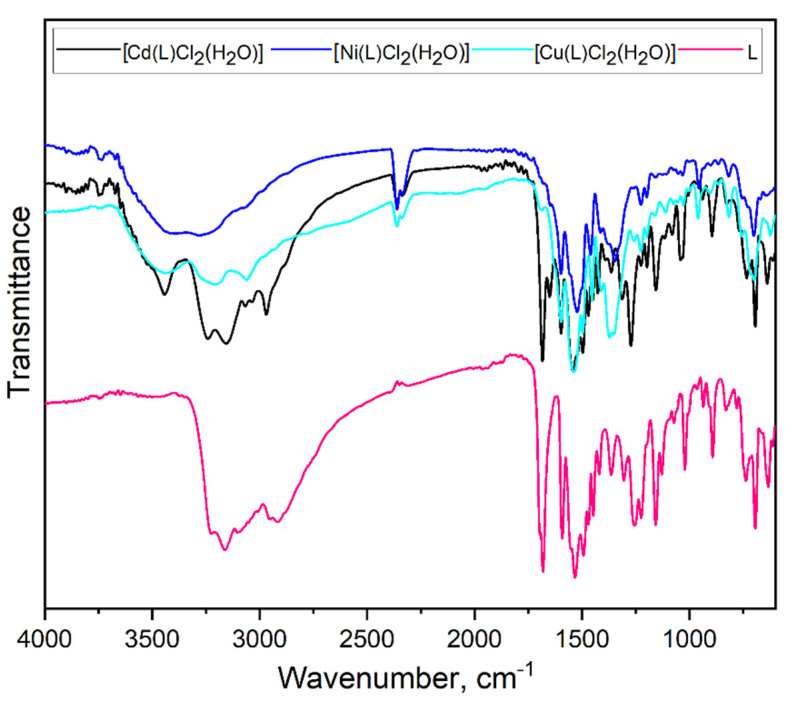
FTIR spectra of pure ligand and obtained complex of [Cd(L)Cl_2_(H_2_O)], [Ni(L)Cl_2_(H_2_O)] and [Cu(L)Cl_2_(H_2_O)].

Figure 4 and Figure 5 present the FTIR spectra of pure ligand and obtained coordination compounds. The spectrum of the pure ligand contains peaks in the region of 3200–2950 cm**^−^**^1^ which are assigned to the *ν*(NH) vibrations [26]. The spectra of [Co(L)Cl_2_], [Ni(L)Cl_2_(H_2_O)], [Cu(L)Cl_2_(H_2_O)] and [Zn(L)Cl_2_] also include slightly moved peaks at the same region. This indicates the presence of an amino group, which does not take part in the process of complex formation. All obtained coordination compounds have new-formed peaks in the range of 3600–3400 cm**^−^**^1^ which can be attributed to the *ν*(OH). In the spectrum of pure ligand, the strong peak at 1681 cm**^−^**^1^ is presented, which corresponds to the *ν*(C=O) presence [27]. In the case of [Co(L)Cl_2_], [Ni(L)Cl_2_(H_2_O)], [Cu(L)Cl_2_(H_2_O)] and [Zn(L)Cl_2_] this peak is very weak or completely absent, indicating the movement of the hydrogen atom to the nearest donor atom, which leads to the formation of the C–OH group.

The uncoordinated ligand shows bands at 1592 cm**^−^**^1^ due to *ν*(C=C), 1532 cm**^−^**^1^ due to *ν*(C=N) and 1021 cm**^−^**^1^ due to *ν*(N-N), respectively. In the case of all achieved complexes, the peak, which correspond to the *ν*(C=N), is shifted toward the higher and lower frequencies regions as a result of coordination between metals ions and organic ligand. This peak indicates that one nitrogen atom is involved in the complexes formation. The vibration modes of *ν*(C=N) and *ν*(N-N) for all coordination compounds are shifted as well. The absorption peaks are observed in the pure ligand at 1253 cm**^−^**^1^ and 802 cm**^−^**^1^ which can be related to the *ν*(C=S). For the following complexes: [Co(L)Cl_2_], [Ni(L)Cl_2_(H_2_O)], [Cu(L)Cl_2_(H_2_O)] and [Zn(L)Cl_2_], these modes are shifted to the higher wavenumber region. This shift indicates the coordination of the metal ions with sulfur atom. For pure ligand, there are also modes of *β*(CH) and *γ*(CH) in the ranges 1370–1300 cm**^−^**^1^ and 890–715 cm**^−^**^1^, respectively. In the complexes they appear in similar ranges.

Thus, from the obtained FTIR spectra we can say that the ligand acts in the bidentate manner for the most cases. The sulfur atom and nitrogen atom are involved in metal (II) coordination in the case of [Co(L)Cl_2_], [Ni(L)Cl_2_(H_2_O)], [Cu(L)Cl_2_(H_2_O)] and [Zn(L)Cl_2_]. For the [Cd(L)Cl_2_(H_2_O)], the metal is coordinated by one nitrogen atom from the pyridine ring, and ligand behaves as a monodentate one.

### 2.3. Magnetic Properties

**Figure 6:**
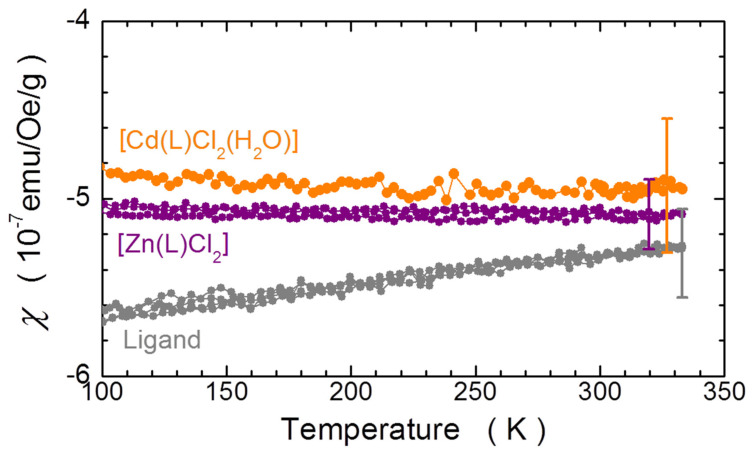
Temperature dependence of the mass magnetic susceptibility *χ* of the coordination compounds [Cd(L)Cl_2_(H_2_O)] (orange) and [Zn(L)Cl_2_] (purple) and of the uncoordinated organic L ligand (gray). The color-matched bars indicate the magnitude of the experimental error bar determined predominantly by the uncertainty of the masses of the samples: 1.5(1), 4.3(1) and 2.5(1) mg for [Cd(L)Cl_2_(H_2_O)], [Zn(L)Cl_2_] and the uncoordinated organic L ligand, respectively.

The magnetic response of the coordination compounds synthesized here is determined by the spin state of the metal ion and by the diamagnetic response of the whole structure of the compound, i.e., the organic ligand, the metal and their coordinate bonds. Although the diamagnetic response of the organic ligand can be established by means of the direct measurements (we present the results in Figure 6), it is of a limited help here since the chemical structures of the final coordination compounds differ in a few key places. We therefore employ the measurements of [Zn(L)Cl_2_] and [Cd(L)Cl_2_(H_2_O)], that is, of the two compounds in which the coordinating metals (Zn and Cd) are expected to retain their closed *d*-shell configuration (3*d*^10^ and 4*d*^10^, respectively). The temperature *T* dependence of the magnetic susceptibility of these two compounds as well as of the organic ligand is presented in Figure 6. Clearly, with the experimental uncertainty, these three compounds exert nearly the same diamagnetic response. Interestingly, however, though the diamagnetism of the ligand is temperature-dependent, both *d*^10^ coordination compounds do not exhibit any temperature dependence of their magnetic susceptibility *χ* within the exercised here temperature range. Moreover, these susceptibilities are found to be nearly the same, or they can be regarded identical within the experimental uncertainty. The latter has been determined predominantly by the uncertainty of the mass of the investigated specimen (specified in the caption to Figure 6). We take, therefore, the value of *χ*_dia_ *= −*5 × 10**^−^**^7^ emu/g/Oe, the average of the two magnitudes of *χ* of Zn and Cd coordinated compounds, as the reference value of the diamagnetic susceptibility for the remaining three coordination compounds investigated in this study.

**Figure 7:**
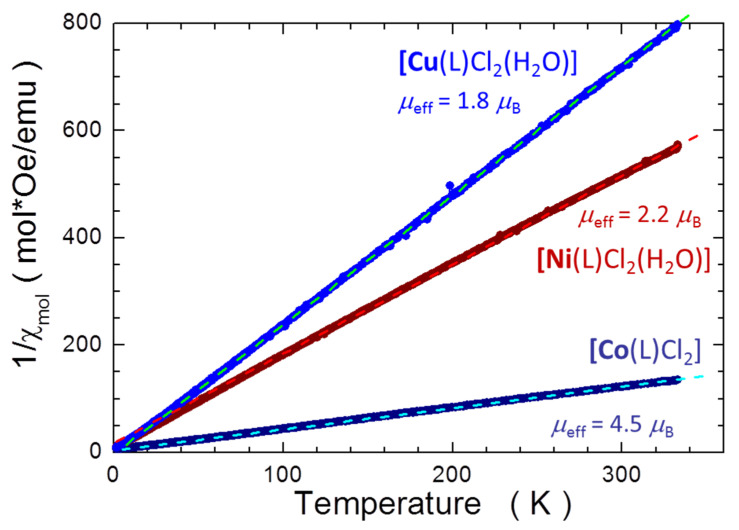
Temperature dependence of the inverse of the molar magnetic susceptibility. χ_m_ of the coordination compounds [Cu(L)Cl_2_(H_2_O)], [Ni(L)Cl_2_(H_2_O)] and [Co(L)Cl_2_] (blue, brown and navy markers, respectively). The slopes of dashed straight lines of corresponding hues define the magnitudes of the Curie constants and so of the effective magnetic moments *μ*_eff_, which values are indicated in the figure.

The remaining three coordination compounds [Co(L)Cl_2_], [Ni(L)Cl_2_(H_2_O)] and [Cu(L)Cl_2_(H_2_O)] contain transition metal group atoms with unfilled 3*d* shells: 3*d*^7^, 3*d*^8^ and 3*d*^9^ for Co, Ni and Cu, respectively. These metals ions very often experience a square planar ligand field. In such coordination, the *d* levels should split into four sets with the *d*_xz_ and *d*_yz_ orbitals forming the lowest levels and the *d*_x2–y2_ orbital as the highest energy one. The electron distribution among these orbitals, or the spin state of the ion, is determined by the ratio of the ligand field splitting Δ_sp_ ≅ 1.74Δ_0_ to the spin pairing energy. This last expression represents the fully symmetric square configuration. Δ_0_ denotes the magnitude of the *e*_g_ to *t*_2*g*_ splitting in the octahedral configuration. In order to gain quantitative information on the spin state of the metal ions in these complexes, we measured the magnetic response as the function of temperature *T* in a broad temperature range, from 2 to 330 K. All compounds exhibit a strong paramagnetic response. The magnetic signal is positive and increases strongly on lowering *T*. The results are presented in Figure 7 in the time honored manner of the Curie–Weiss plot, that is, we plot the inverse of the molar susceptibility *χ*_m_ of the all three metals as the function of *T*. To establish **_χ_**_m_, the experimental data are corrected for the value of the **_χ_**_dia_ established previously for *d*^10^ complexes with the same organic ligand (Figure 6). The results form convincingly straight lines. The slopes of these lines yield the magnitudes of the inverse of the Curie constant *C*_mol_**^−^**^1^ = 3 *k*_B_**/***μ*_eff_^2^**/***N*_A_, from which the magnitude of the effective magnetic moment *μ*_eff_^2^ ≅ 8**/***C*_mol_ is readily obtained. In this notation *μ*_eff_ is given in units of Bohr magnetons μ_B_, *k*_B_ is the Boltzmann constant and *N*_A_ is the Avogadro number. The magnitudes of *μ*_eff_ established upon this procedure are given in the Figure 7. We find that the *μ*_eff_ of Cu in [Cu(L)Cl_2_(H_2_O)] matches the expected values for a typical Cu^2+^ ion. For the 3*d*^9^ configuration with a fully quenched orbital momentum *L* the total angular orbital momentum *J* assumes the magnitude of spin moment *S* = ½ and so *μ*_eff_ should be 1.73 μ_B_. Experimentally, a value of about 1.9 was found [28], and our measurements yielded *μ*_eff_^Cu^ = 1.8 μ_B_. This indicates that presence of water molecules in this coordination compound does not really alter the distribution of *d* orbitals in Cu, similarly to *d*^10^ ions in [Zn(L)Cl_2_] and [Cd(L)Cl_2_(H_2_O)]. The data collected for the [Co(L)Cl_2_] yield *μ*_eff_^Co^ = 4.5 μ_B_. This value is somehow above the *L*-quenched theoretical value of 3.8 μ_B_ and very close to the experimental one of 4.8 μ_B_. The latter appears to be a well-known exception of Co^2+^ ions across the whole 3*d* series, where an orbital contribution pushes *μ*_eff_ above the spin-only value [28]. This finding indicates that also in this case the ligands coordination does not alter the high spin state of Co^2+^ ions in [Co(L)Cl_2_]. In the case of [Ni(L)Cl_2_(H_2_O)], the magnitude of the experimental Curie constant yields *μ*_eff_^Ni^ = 2.2 μ_B_. This value lies significantly below both the theoretically expected and experimental magnitudes of 2.83 μ_B_ and 3.2 μ_B_, respectively [28]. However, such anomalous magnetic behaviors of some nickel and other transition metal complexes have already been reported several times in the literature [29,30,31,32,33,34,35,36].

### 2.4. ADMET

**Figure 8:**
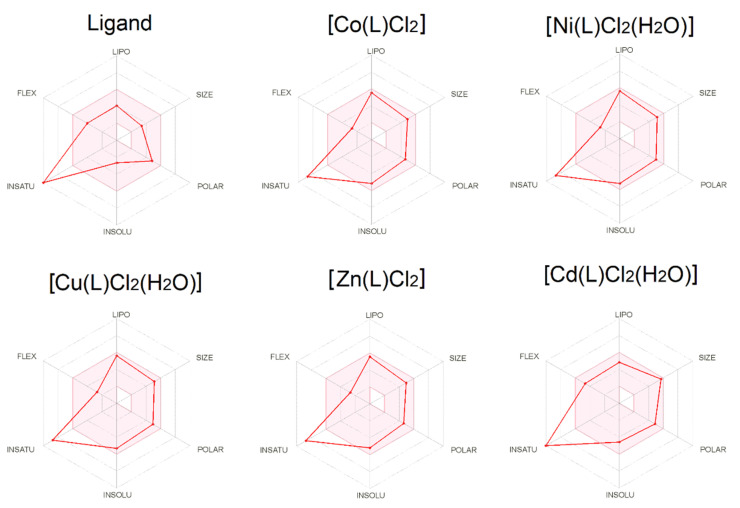
Oral bioavailability graphs generated using the SwissADME service. The red–colored zone is physicochemically suitable for oral bioavailability. LIPO—lipophility (−0.7 < XlogP3 < +5.0); SIZE—molecular weight (150 g/mol < MW < 500 g/mol); POLAR—polarity (20 Å^2^ < TPSA < 130 Å^2^); INSOLU—insolubility (0 < logS < 6); INSATU—insaturation (0.25 < fraction Csp3 < 1); FLEX—flexibility (0 < num. of rotatable bonds < 9).

ADME analysis enables the first selection of compounds for costly biological studies. The selected compounds should meet the basic properties that will suggest their usefulness in research on a specific organ. All tested complexes meet the rules of Lipinski [37], Ghose [38], Egan [39], Veber [40] and Muegge [41]. Bioavailability Radars (Figure 8) present six physicochemical properties such as lipophilicity, size, polarity, solubility, flexibility and saturation. All compounds have a good bioavailability score of 0.55, calculated from relying on total charge, TPSA, and violation to the Lipinski filter. All tested compounds are good drug candidates. 

**Figure 9:**
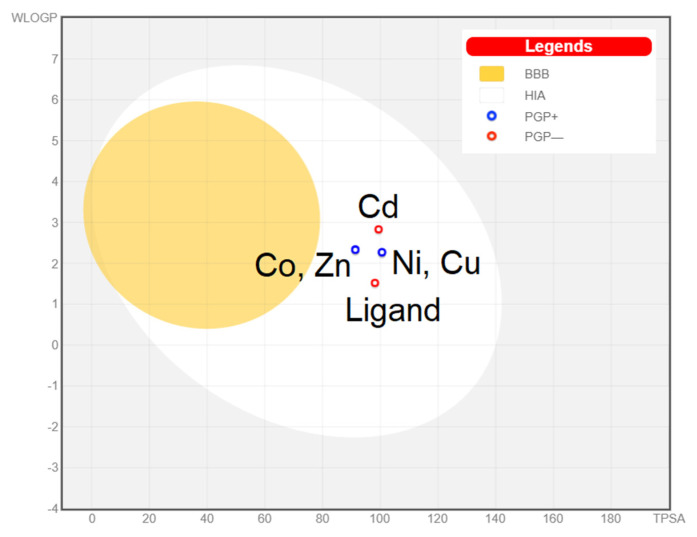
BOILED-Egg diagram for all studied compounds.

Figure 9 presents the BOILED-Egg diagram for the studied compounds. All of them are absorbed from the gastrointestinal tract (all compounds can be found in the white area of the diagram) and do not pass the blood–brain barrier (none of them can be found in the yellow area). Cadmium complex is not P-gp substrate that can be a candidate against multidrug-resistant cancer cells. The more negative the log Kp value, the less the molecule passes through the skin. All compounds displayed negative values ranging from −7.08 to −5.64 cm/s, meaning that they are not accessible through the skin. All compounds belong to the fourth class of toxicity (harmful if swallowed; 300 < LD_50_ ≤ 2000). Predicted LD_50_ doses: ligand—1700 mg/kg; [Co(L)Cl_2_]—1000 mg/kg; [Ni(L)Cl_2_(H_2_O)]—1000 mg/kg; [Cu(L)Cl_2_(H_2_O)]—1600 mg/kg; [Zn(L)Cl_2_]—1600 mg/kg; [Cd(L)Cl_2_(H_2_O)]—400 mg/kg.

### 2.5. Cytotoxicity Assay

The inhibitory potency of the newly prepared compounds was tested using the colorimetric MTT assay. The concentrations of the compounds that induced 50% inhibition of cell viability (IC_50_) are tabulated in Table 2.

**Figure 10:**
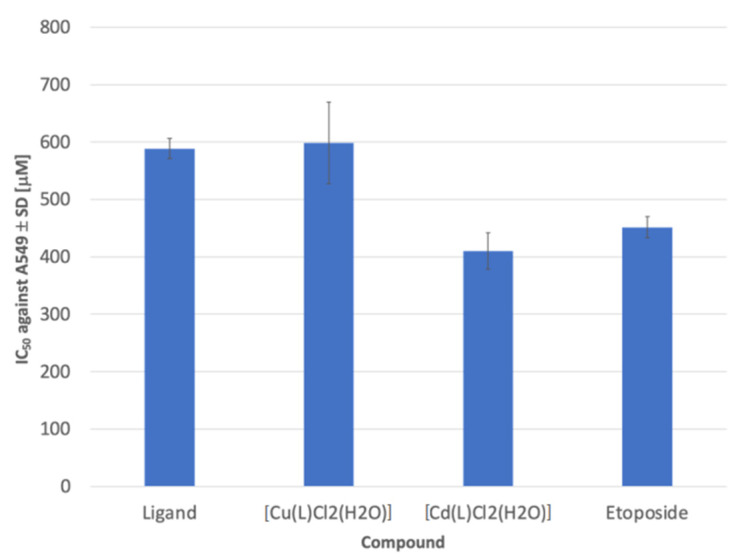
In vitro cytotoxic activity of the three most potent compounds and etoposide as reference material. Statistical significance was assessed using one-way ANOVA analysis. *p* < 0.01 was considered as significantly different between cytotoxicity of etoposide and the best tested compounds.

**Figure 11:**
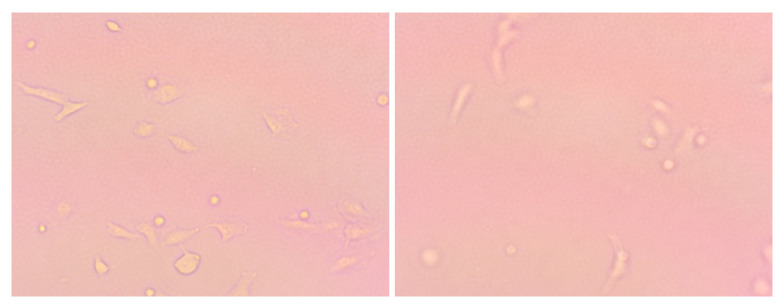
Antitumor effect of [Cd(L)Cl_2_(H_2_O)] compound on A549 cancer cells. Cells were cultured in the absence (**left**) or in the presence (**right**) of the tested compound. Representative phase-contrast cell images are shown after 24 h (100× magnification).

Significantly, three of the tested derivatives displayed potent cytotoxic activity against A549 cell lines of IC_50_ range 410–599 μM (Figure 10). On the other hand, while comparing the resultant activities of the compounds with etoposide, compound [Cd(L)Cl_2_(H_2_O)] represented more potent activity than that of the reference drug against A549 cell lines (Figure 11). A mild decrease in the activity was determined by [Cu(L)Cl_2_] compound to be approximately equipotent to the free ligand.

## 3. Materials and Methods

### 3.1. Synthesis of the L Ligand

For the first time, this compound was obtained by our team by heating phenyl isocyanate and pyridin-3-carboxylic acid hydrazide in an oil bath at 100 °C for 8 h [42]. Here, we present the modified method of the synthesis—we used methanol as the solvent and reduced the heating time to 1 h. The structure and the purity of the compound were confirmed by determining the melting point and the ^1^H and ^13^NMR spectroscopy.

Pyridin-3-carboxylic acid hydrazide (1.37 g, 0.01 mole) was dissolved in methanol (40 mL), and phenyl isothiocyanate (1.35 g, 0.01 mole) was added. The mixture was heated at the reflux temperature of the solvent for 1 h. The mixture was then cooled, and the precipitate was crystallized from ethanol.

**Ligand L (C_13_H_12_N_4_OS)** (272.32 g/mol), (yield 93%), (m.p. 174–176 °C), ^1^H NMR (DMSO-d_6_) δ: 7.14–7.57 (m, 5H, CH_phenyl_), 8.26–8.30 (d, 1H, CH_pyridin_), 8.74 (s, 1H, CH_pyridin_), 8.76 (s, 1H, CH_pyridin_), 9.76 (s, 1H, NH), 9.87 (s, 1H, NH), 10.77 (s, 1H, NH); ^13^C NMR (DMSO-d_6_) δ: 122, 125, 126, 128, 130, 139, 140, 164, 181. FTIR spectra (KBr, cm^−1^): *ν*(NH) 3162, 2916; *ν*(C=O) 1682; *ν*(C=C) 1592; *ν*(C=N) 1532; *δ*(NH) 1494; *β*(CH) 1363, 1305; *ν*(C=S) 1253, 802; *ν*(N-N) 1021; *γ*(CH) 737, 693.

### 3.2. Synthesis of the Coordination Compounds

In each case, 162 mg (0.6 mmol) of the ligand was dissolved in the ethanol at 50 °C with constant stirring, until the clear ligand solutions were obtained. These solutions were then added to water/ethanol (*v/v* = 1/1) solutions of 0.6 mmol of Co(II), Ni(II), Cu(II), Zn(II) and Cd(II) chlorides. Total volumes did not exceed 60 mL. Reaction mixtures were then stirred on a magnetic stirrer for 4 h. After that time, the obtained precipitates of complexes were filtered, washed several times with ethanol and water/ethanol (*v/v* = 1/1) and later dried in the open air, weighted and analyzed.

**[Co(L)Cl_2_] (C_13_H_12_N_4_OSCoCl_2_)** (402.16 g/mol), (yield 46%), anal. calculated (%): Co, 14.66; S, 7.97. Found (%): Co, 14.13; S, 8.10. FTIR spectra (KBr, cm^−1^): *ν*(NH) 3197, 3041; *ν*(C=C) 1581; *ν*(C=N) 1550; *δ*(NH) 1495; *β*(CH) 1373, 1322; *ν*(C=S) 1271, 810; *ν*(N-N) 1053; *γ*(CH) 752, 701.

**[Ni(L)Cl_2_(H_2_O)] (C_13_H_12_N_4_OSNiCl_2_)** (401.92 g/mol), (yield 38%), anal. calculated (%): Ni, 14.60; S, 7.98. Found (%): Ni, 14.83; S, 7.67. FTIR spectra (KBr, cm^−1^): *ν*(NH) 3280, 2975; *ν*(C=C) 1598; *ν*(C=N) 1522; *δ*(NH) 1460; *β*(CH) 1414, 1347; *ν*(C=S) 1261, 818; *ν*(N-N) 955; *γ*(CH) 753, 700.

**[Cu(L)Cl_2_(H_2_O)] (C_13_H_12_N_4_OSCuCl_2_)** (406.78 g/mol), (yield 36%), anal. calculated (%): Cu, 15.62; S, 7.88. Found (%): Cu, 15.95; S, 7.50. FTIR spectra (KBr, cm^−1^): *ν*(NH) 3210, 3060; *ν*(C=C) 1596; *ν*(C=N) 1537; *δ*(NH) 1497; *β*(CH) 1372, 1350; *ν*(C=S) 1258, 815; *ν*(N-N) 960; *γ*(CH) 756, 698.

**[Zn(L)Cl_2_] (C_13_H_12_N_4_OSZnCl_2_)** (408.64 g/mol), (yield 63%), anal. calculated (%): Zn, 16.01; S, 7.85. Found (%): Zn, 15.83; S, 8.00. FTIR spectra (KBr, cm^−1^): *ν*(NH) 3195, 3032; *ν*(C=C) 1612; *ν*(C=N) 1552; *δ*(NH) 1497; *β*(CH) 1377, 1321; *ν*(C=S) 1273, 825; *ν*(N-N) 957; *γ*(CH) 753, 702.

**[Cd(L)Cl_2_(H_2_O)] (C_13_H_14_N_4_O_2_SCdCl_2_)** (473.66 g/mol), (yield 46%), anal. calculated (%): Cd, 23.73; S, 6.77. Found (%): Cd, 23.21; S, 6.20. FTIR spectra (KBr, cm^−1^): *ν*(NH) 3241, 3155; *ν*(C=O) 1684; *ν*(C=C) 1600; *ν*(C=N) 1541; *δ*(NH) 1497; *β*(CH) 1363, 1313; *ν*(C=S) 1272, 826; *ν*(N-N) 1032; *γ*(CH) 767, 693.

### 3.3. Chemistry

All chemicals used for the synthesis were purchased from Sigma-Aldrich (St. Louis, MO, USA), AlfaAesar (Haverhill, MA, USA) and POCH (Gliwice, Poland) companies and used without further purification. Melting points were determined using Fisher–Johns block and presented without corrections. The ^1^H and ^l3^C NMR spectra were recorded on a Bruker Avance 300 spectrometer (Bruker BioSpin GmbH, Rheinstetten, Germany) in d_6_-DMSO. Chemical shifts were reported in parts per million (ppm, δ scale) relative either to internal standard (TMS) or residual solvent peak. The contents of Co(II), Ni(II), Cu(II), Zn(II), Cd(II) and S in solid complexes were determined by the ICP spectroscopy. Standard solutions from Merck (1000 mg/L, Darmstadt, Germany) were used for the preparation of diluted solutions used for calibration. For analysis, distilled water (electrical conductivity 0.05 µS) was used (obtained with Polwater system). Solid samples were decomposed using the Anton Paar Multiwave 3000 closed system instrument. Mineralization was carried out for 45 min at 240 °C under pressure 60 bar. Thermogravimetric analysis of formed complexes was investigated on a Netzsch TG 209 apparatus (NETZSCH GmbH, Selb, Germany) under air atmosphere, v = 20 mL·min^−1^, using ceramic crucibles. Thermograms represent TG-DTG curves in the range of temperature 25 to 800 °C at a heating rate of 10 °C min^−1^. FTIR spectra were performed using IR Tracer-100 Schimadzu Spectrometer (Schimadzu Corporation, Kyoto, Japan) (4000–600 cm^−1^) with an accuracy of recording of 1 cm^−1^, using KBr pellets.

### 3.4. ADMET 

Compounds were analyzed by using SwissADME service (Swiss Institute of Bioinformatics 2021) [43,44] and ProTOX II service [45] to obtain computational pharmacokinetic and toxicologic profiles.

### 3.5. Cytotoxicity Assay

In order to survey the antilung cancer effects of the new synthesized compounds, *3-(4,5-dimethylthiazol-2-yl)-2,5-diphenyl-2H-tetrazolium bromide* (MTT) assay was used on human lung carcinoma cell lines (A549) [46,47]. Cell lines were maintained in a Dulbecco modified eagle medium (DMEM) with 10% Fetal bovine serum (FBS) and 1% penicillin/streptomycin antibiotic. Cytotoxic activity screening was performed using MTT assay. Cells were placed in 104 cells/well for 24 h, and all cells were cultivated at 37 °C and 5% CO_2_. After incubation for 24 h, the cells were placed into 96 well plates, and different concentrations of sample (10, 50, 100, 200, 400 and 600 μM) were added; the incubation was continued for 24 h. The A549 cell lines were incubated with 50 μL of MTT at 37 °C for 2 h. After incubation, media were removed slowly, and we added 100 μL of dimethyl sulfoxide to each well to dissolve insoluble formazan crystals; they were then incubated for 10 min. The cytotoxicity was estimated by IC_50_ in (μM), and the concentration that inhibits 50% of growth of cancer cell from the absorbance values was recorded at 570 nm.

### 3.6. Magnetic Measurements

Magnetic measurements were performed in a superconducting quantum interference device (SQUID)-based magnetometer MPMS XL of a Quantum Design (San Diego, CA, USA). In order to facilitate accurate measurements of single-milligram-sized powder specimens, two experimental approaches were applied and compared. In the first one, the powders are mixed with a strongly ethanol-diluted GE-varnish, and such thick solutions are transferred onto small rectangular silicon 5 × 4 × 0.2 mm^3^ plates to provide a solid support and to ease the handling, as already exercised and validated [48,49]. The relatively weak magnetic signal of the Si base plate has been adequately removed from the results, yielding the magnetic response of the investigated substances. The need for the ex situ compensation of the signal exerted by the specimen carriers is alleviated by the second approach in which an in situ compensation method of the specimen carriers (polycarbonate capsules), recently elaborated by some of the present authors, is applied [50]. We confirm that within the uncertainty of the exact mass of the specimens used in each of the measurements (±0.1 mg), both methods yield the same results. Regardless to the approach to the measurement, the data reduction and the final magnetic moment determination were performed following the code elaborated for high sensitivity studies of minute magnetic signals [51].

## 4. Conclusions

In the current years, numerous thiosemicarbazide derivatives have been synthesized and investigated with regard to their biological activity. In this article, thiosemicarbazide-based organic ligand and five metal (II) complexes were synthesized, and their physicochemical and biological properties were investigated. All six compounds form solids stable under room temperature. When heated, they gradually decompose. The final solid products of decomposition of the studied coordination compounds are corresponding metal (II) oxides. The Fourier-transform infrared spectroscopy spectra revealed the presence of water molecules in Ni, Cu and Cd complexes. Furthermore, the FTIR results were corroborated by the magnetic measurements. The quantitative information on the spin state of the metal ions measured as the function of temperature (from 2 to 330 K) has shown that Co, Ni and Cu compounds exhibit strong paramagnetic response. The magnetic signal is positive and increases strongly on lowering temperature. Interestingly, the diamagnetism of the ligand is temperature-dependent, but both Zn and Cd compounds did not exhibit any temperature dependence of their magnetic susceptibility within the exercised temperature range. The ADME/Tox analysis of the new synthesized organic ligand and coordination compounds indicated that all compounds do not violate the Lipinski, Ghose, Egan, Veber and Muegge rules. Additionally, all compounds have good bioavailability score of 0.55, and all belong to the fourth class of toxicity. New compounds were evaluated as cytotoxic candidates against human lung carcinoma cells (A549), with etoposide serving as a reference material. The compounds we have developed show comparable anticancer properties to etoposide. Organic ligand exhibited potent anticancer activity (with IC_50_ = 589 ± 18 μM), and [Cu(L)Cl_2_] complex was equipotent to it (with IC_50_ = 599 ± 71 μM). There are several treatment options for small cell lung cancer involving etoposide as a topoisomerase inhibitor. We used etoposide as a reference due to its frequent use. The investigated compounds exhibit comparable antitumor properties to this reference, as outlined above. However, one of them, [Cd(L)Cl_2_(H_2_O)], has better biological properties (IC_50_ = 410 ± 31 μM). The antitumor activity may be related to the type of metabolism of the compound inside the cells. This may also apply to cellular pathways that are a therapeutic target [52]. The full mechanism of action of the complexes themselves remains unknown. In this regard, research in the field of molecular biology will be continued. It is, however, known that free cadmium affects several processes in cells. This includes: cells proliferation, cells differentiation, apoptosis, DNA repair and production reactive oxygen species [53,54]. Such studies are of a great importance, taking into consideration the search for new drugs and thus the possibility of developing new treatment possibilities. This gives hope for a more effective fight against one of the most dangerous types of cancer.

## Figures and Tables

**Table 1:** Thermal decomposition data of the ligand and Co(II) Ni(II) Cu(II) Zn(II) and Cd(II) complexes.

Compound	Stages	Temperature Range				Final Solid Product of the Thermal Decomposition
		I	II	III	IV	
Ligand	3	180–210	210–400	550–700	-	-
[Co(L)Cl_2_]	3	275–460	460–550	550–640	-	CoO
[Ni(L)Cl_2_(H_2_O)]	4	40–120	120–250	250–420	420–600	NiO
[Cu(L)Cl_2_(H_2_O)]	4	40–150	150–250	250–380	380–720	CuO
[Zn(L)Cl_2_]	2	280–500	500–800	-	-	ZnO
[Cd(L)Cl_2_(H_2_O)]	3	40–200	210–450	450–760	-	CdO

**Table 2:** In vitro cytotoxic efficiency of the new derivatives against A549 (IC_50_ values = mean ± SD of two independent determinations; IC_50_—50% inhibition of the cell viability, (μM)).

Compound	IC_50_ against A549 ± SD (μM)
Ligand	589 ± 18
[Co(L)Cl_2_]	>600
[Ni(L)Cl_2_(H_2_O)]	>600
[Cu(L)Cl_2_(H_2_O)]	599 ± 71
[Zn(L)Cl_2_]	>600
[Cd(L)Cl_2_(H_2_O)]	410 ± 31
Etoposide	452 ± 18

## Data Availability

Not applicable.

## Sample Availability

Samples of the compounds are available from the authors.
